# Supplementary feeding of wild birds indirectly affects ground beetle populations in suburban gardens

**DOI:** 10.1007/s11252-014-0404-x

**Published:** 2014-08-22

**Authors:** Melanie E. Orros, Rebecca L. Thomas, Graham J. Holloway, Mark D. E. Fellowes

**Affiliations:** School of Biological Sciences, Harborne Building, University of Reading, Reading, RG6 6AS UK

**Keywords:** Carabidae, Ground beetles, Garden birds, Small mammals, Urban ecology, Wild bird feeding

## Abstract

Supplementary feeding of wild birds by domestic garden-holders is a globally widespread and popular form of human–wildlife interaction, particularly in urban areas. Vast amounts of energy are thus being added to garden ecosystems. However, the potential indirect effects of this activity on non-avian species have been little studied to date, with the only two previous studies taking place under experimentally manipulated conditions. Here we present the first evidence of a localised depletive effect of wild bird feeding on ground beetles (Coleoptera: Carabidae) in suburban gardens under the usual feeding patterns of the garden-holders. We trapped significantly fewer ground beetles directly under bird-feeding stations than in matched areas of habitat away from feeders. Video analysis also revealed significantly higher activity by ground-foraging birds under the feeding stations than in the control areas. Small mammal trapping revealed no evidence that these species differ in abundance between gardens with and without bird feeders. We therefore suggest that local increases in ground-foraging activity by bird species whose diets encompass arthropods as well as seed material are responsible for the reduction in ground beetle numbers. Our work therefore illustrates that providing food for wild birds can have indirect negative effects on palatable prey species under typical conditions.

## Introduction

Providing food for wild birds in domestic gardens is a popular activity across many parts of the world. In the UK, estimates of participation from large-scale, relatively unbiased data sets are 56 % of English households (ODPM 2003) and 48 % of UK ones (Davies et al. 2009). Similar household estimates exist for Australia (36–48 %; Ishigame and Baxter 2007) and the USA (47 %; calculated from US Fish and Wildlife Service 2006, and US Census Bureau 2011). Alternative indicators are the US$3.4 billion spent by US residents on wild bird food in 2006 (US Fish and Wildlife Service 2006) and the mean densities of bird feeders in England, which range from 15 to 560 km^−2^ amongst counties (Fuller et al. 2012). Whatever the viewpoint, although to our knowledge no calorific estimates have yet been published, it is clear that vast amounts of energy are being added to ecosystems, most often in urbanised areas.

Despite the evident scale of wild bird feeding by private households, little research has been undertaken into its ecological consequences within domestic gardens (but see e.g., Cowie and Hinsley 1988a; Cowie and Simons 1991). Furthermore, studies have typically considered the direct or indirect effects on birds themselves (e.g., Jansson et al. 1981; Robb et al. 2008; Orros and Fellowes 2014) or the socioeconomic factors associated with those who feed (e.g., Fuller and Irvine 2010; Fuller et al. 2012). The potential wider influences on other species have been virtually ignored to date. This is particularly surprising given that many bird taxa are key predators, often with broad diets (Capinera 2010). For example, many bird species that readily feed on supplementary food in gardens often selectively provision young with ‘natural’ foods, such as arthropods (e.g., Cowie and Hinsley 1988b).

We are aware of just two examples in the scientific literature of the indirect effects of wild bird feeding on non-avian taxa, both involving arthropods. Numbers of mealworms (*Tenebrio molitor*; used to mimic overwintering bark-dwelling arthropods) in woodland plots with bird feeders were reduced relative to control plots without feeders (Martinson and Flaspohler 2003). In the other example, the size and survival of exposed aphid colonies placed close to domestic garden bird feeders were reduced compared with protected colonies at the same distance from the feeders (Orros and Fellowes 2012). No such differences were found in control gardens without bird feeders. Both sets of authors attributed their findings to higher levels of avian predation of the target arthropod species around bird feeders because of localised increases in bird densities. However, given that both were manipulative experiments with the prey arthropods artificially introduced and the type and location of the bird feeders controlled, it is unclear whether such detectable effects of supplementary feeding of wild birds are also observed in natural arthropod populations.

Although further examples are lacking, it is possible to draw parallels with effects seen around bird nesting sites as both feeders and nestlings can represent fixed central point attractants (repeated visits by the same birds to nest sites vs. a variety to feeders; see Orros and Fellowes 2012). In this context, depletion of unmanipulated, wild arthropods has been observed in areas with avian nest boxes compared with sites without (Sanz 2001), and around colonial and solitary avian nesting sites (e.g., Jäntti et al. 2001; Bonal and Aparicio 2008).

The discoveries of depletive effects of wild bird feeding on arthropods with different lifestyles (bark- and plant-dwelling) and in different habitats (woodland and urban gardens) hint that such effects could occur amongst other arthropod taxa. Further, the similar findings around other avian point attractants provide an indication that such effects may occur in ‘real-world’ settings without experimental manipulation. We therefore designed a study to investigate whether ground-dwelling arthropods might also be subject to similar depletion around domestic garden bird feeders and whether a nonmanipulative approach would detect this.

At ground level, terrestrial mammals with diets including plant and invertebrate material may also be attracted to forage under bird feeders and also predate upon ground-living arthropods. Therefore, such species could potentially contribute to or even be the primary factor behind any depletion in arthropod abundance. Our scope therefore also encompassed various small rodent species (hereafter ‘small mammals’). These taxa are known to use UK gardens and have diets that overlap to varying extents with those of feeder-using birds (Buczacki 2007; Churchfield 2008; Gurnell and Hare 2008).

We selected ground beetles (Coleoptera: Carabidae) as a model ground-living arthropod taxon in order to examine possible depletion under bird feeders because many species are predated upon by birds and mammals, common in UK gardens and relatively easily identifiable to species level (Thiele 1977; Luff 2007; Gurnell and Hare 2008; Capinera 2010).

We hypothesised that (1) ground-foraging birds are more likely to search for food in areas underneath suspended bird feeders than in similar areas away from feeders; (2) that the abundance of small mammals would be higher in gardens in which bird food was supplied than those without; and (3) that as a result of such increases in vertebrate predator numbers we would see a local reduction in the abundance and perhaps diversity of ground beetles under bird feeders.

## Methods

Our broad scope led us to use two different spatial scales in order to encompass all of the above-mentioned taxa. Carabidae and ground-feeding birds were examined within gardens because the highly mobile point-feeding nature of birds attracted to feeding stations (and the resources below) makes it possible to examine differences at a fine spatial scale. We used matched pairs of habitat with and without bird feeders within individual gardens. A between-garden spatial scale was adopted for our target mammal taxa because small mammal home ranges generally greatly exceed individual UK garden sizes [e.g., *c.* 3,800 m^2^ for bank voles (*Myodes glareolus*), 4,300 m^2^ for wood mice (*Apodemus sylvaticus*), vs mean UK garden size of *c.* 190 m^2^ (Davies et al. 2009)].

### Study sites

The study took place in suburban gardens within and around Reading, a large town in Berkshire, southern England [51°27′N, 0°58′W population: 156 000 (Office for Statistics 2013)] from July to September 2012. Garden owners were recruited by means of approaches to relevant local groups and local media (radio, newspapers, website) and to participants in previous studies by the authors. Gardens in the study reflect southern English suburbia. Gardens varied in size from approximately 40 m^2^ to over 120 m^2^. All gardens were at least 50 % grassed lawn (large gardens had a greater proportion under lawn) and all had some tree/shrub cover. For the within-garden spatial-scale work, 28 bird-feeding households that had fed birds for at least 1 year were recruited. The between-garden element utilised a separate group of 36 households, half that regularly fed birds and half that did not feed. All bird-feeding households were asked to continue their usual pattern of feeding to allow us to look for observable effects under typical conditions.

### Study design

#### Bird activity

Diurnal bird activity on the ground around both sets of pitfall traps (see Carabidae abundance and diversity below) was recorded using motion-sensitive video cameras (HandyKam, Cornwall, UK and Bushnell, Surrey, UK) in six gardens in the within-garden part of the study. These gardens were selected according to householder preference and security considerations. Cameras recorded for 20 s after motion for a 24-h period during the September carabid-trapping session. Gardens were not filmed simultaneously or in all survey sessions because of equipment availability. Cameras were set for 24 h to capture the equivalent of a full daylight cycle. A pilot study indicated that night-time recording was insufficiently reliable with the equipment available and therefore only daylight footage was used.

The species and the start time and duration were recorded for each observation. An activity score (total number of seconds for which a species was seen around the pitfall area) was calculated for each species for the control and feeder sites because individuals could not be distinguished. The species scores for each site do not sum to the total time over which activity was observed because of simultaneous visits.

#### Small mammal abundance and diversity

Twenty live traps [10 Longworth and 10 TubeTrap (BioEcoSS Ltd, UK)] were used in each of the 36 gardens in the between-gardens part of the study for five consecutive nights between August and September. Six gardens were surveyed simultaneously over each five-night period.

Traps were placed in a standardised pattern at 3-m intervals adjacent to the garden periphery in areas of cover. Traps contained hay and were baited with mixed seed, cat food and apple and set for a maximum of 12 h overnight following recommended practice (Gurnell and Flowerdew 2006). Prior to release, trapped mammals were identified to species and individually marked by fur clipping to avoid multiple counting.

#### Carabidae abundance and diversity

Pitfall traps were installed in all gardens in the within-garden part of the study in two sets of three, one set directly under a bird-feeding station (hereafter the ‘feeder traps’) and the second set (hereafter the ‘control traps’) in a matched habitat (e.g., lawn or flowerbed) with similar levels of human disturbance situated as far as possible from the feeding station (range *c.* 4–15 m).

The three traps within each set were sited within 0.3 m of each other. Traps were disposable plastic drinks cups (6.6 cm diameter, 0.2 L capacity) dug in to ground level and one-third filled with an oversaturated salt solution with a drop of unscented washing-up liquid (Surcare, McBride, UK) to reduce surface tension. We used 9.5-cm-diameter plastic plant saucers as covers to provide shelter from rain and restrict vertebrate access. These were fixed *c.* 2 cm above the cup using plant stakes through two holes drilled in the sides of the saucer.

Salt solution was used because of the safety implications of using ethylene glycol in areas accessible by children and pets (Hall 1991; Lemieux and Lindgren 1999; Woodcock 2005) and the significant cost of the less toxic alternative, propylene glycol, for large-scale studies. Furthermore, many manufacturers advise that propylene glycol should not contaminate soil or watercourses (e.g., ReAgent, Cheshire, UK; http://www.reagent.co.uk/uploads/documents/PROPANEDIOL-TECH-MSDS.pdf), a considerable risk in countries with relatively high rainfall such as the UK. Although evaporation can be a problem with salt solution, preliminary testing revealed that this was not an issue in our study climate. Oversaturation with an additional heaped teaspoon of salt negated the risk of the solution becoming unsaturated (and therefore reducing preservation effectivity) during heavy rainfall.

Traps were set for 7 days 4 weeks apart in early July, August and September. On the seventh day of each session, all contents were transferred to the laboratory to be cleaned and sorted. Carabids were stored in 70 % ethanol until identification under a stereomicroscope (Nikon, Surrey, UK) using Luff (2007).

### Statistical analyses

Small mammal abundance was analysed using R (v. 2.12.0, R Core Development Team 2010). Minitab was used for all other analyses (v. 16; Minitab, Inc., State College, PA). Data were checked for normality using Anderson–Darling tests prior to analysis. As a result, Carabidae species number was square-root transformed.

#### Birds

Owing to the small sample size and non-Normal distribution, the bird activity data were analysed using Wilcoxon matched-pairs signed-ranks tests to compare the activity scores and number of species between feeder and control trap areas.

#### Small mammals

For small mammal abundance, Wilcoxon rank sum tests were performed on the total numbers trapped (species counts pooled to increase power) as the assumptions of generalised linear modelling were not met with either quasi-Poisson or negative binomial error distributions. Species diversity was not investigated statistically as most gardens in which mammals were trapped had only a single species (see Results).

#### Carabidae

Gardens were excluded from all analyses if no carabids were trapped. Power analyses were performed for each month and subsequently data were pooled in the analyses presented here.

Paired two-tailed *t*-tests were carried out to test for differences between the feeder and control traps in total numbers of individuals, the total number of the most common species (*Pterostichus madidus*), numbers of all other Carabidae combined and the total number of carabid species. Non-parametric Wilcoxon matched-pairs signed-ranks tests were also carried out on the latter two differences owing to low power.

## Results

### Birds

Nine bird species were recorded in the feeder trap areas across the six gardens, with woodpigeon (*Columba palumbus*) making up almost half (47.8 %) of total activity (Table 1). Activity in the control trap areas was significantly lower [*P* = 0.036; Wilcoxon statistic = 21.0; median difference = 1,344 s; interquartile range (Q1–Q3) = 693–8,937]; just 46 s for blackbird (*Turdus merula*) in one garden. The number of species observed in the feeder areas was also significantly higher than in the control areas (*P* = 0.036; Wilcoxon statistic = 21.0; median difference = 5.00; Q1–Q3 = 4.75–6.75).

**Table 1:** Activity scores (s) of birds recorded under wild bird-feeding stations (feeder areas) and in similar areas of habitat away from feeding (control areas) in suburban domestic gardens in southern England

Species	Activity score (s) per garden						Total activity score (s)
	A	B	C	D	E	F	
Feeder areas							
Woodpigeon (*Columba palumbus*)	7,942	90	3,150	–	–	711	11,893
Blackbird (*Turdus merula*)	246	53	2,770	173	920	–	4,162
Feral pigeon (*Columba livia*)	3,373	211	–	–	37	–	3,621
Dunnock (*Prunella modularis*)	170	–	1,403	780	463	31	2,847
Chaffinch (*Fringilla coelebs*)	1,148	–	–	–	–	–	1,148
Magpie (*Pica pica*)	547	185	–	–	–	–	732
Robin (*Erithacus rubecula*)	43	4	103	162	147	4	463
Collared dove (*Streptopelia decaocto*)	–	–	–	–	–	12	12
Blue tit (*Cyanistes caeruleus*)	1	–	–	4	2	–	7
Control areas							
Blackbird	–	46	–	–	–	–	46

Activity score per garden is the total time in seconds for which the species was observed. Each garden was videoed for 24 h. These values do not sum to the total activity time across all gardens because multiple individuals were observed simultaneously in some videos

Letters represent individual gardens

### Small mammals

Small mammals were trapped in eight feeding and nine non-feeding gardens, with a single species in all but two gardens. Wood mice (*Apodemus sylvaticus*) were by far the most common (*N* = 25 and 29 in feeding and nonfeeding gardens, respectively), with a yellow-necked mouse (*Apodemus flavicollis*) and two bank voles (*Myodes glareolus*) also trapped (Table 2). There was no significant difference in the total numbers between feeding (median = 0; Q1–Q3 = 0.00–2.25) and non-feeding (median = 0.5; Q1–Q3 = 0.00–3.00) gardens (*P* = 0.959; Wilcoxon statistic = 164).

**Table 2:** Total numbers of small mammals trapped in suburban domestic gardens in southern England provisioned with supplementary wild bird food and gardens without such provisioning

Species	Numbers trapped	
	Bird-feeding gardens	Non-feeding gardens
Wood mouse (*Apodemus sylvaticus*)	25	29
Yellow-necked mouse (*Apodemus flavicollis*)	1	0
Bank vole (*Myodes glareolus*)	0	2
Total	26	31
Median number trapped per garden	0	0.5
Range in numbers trapped amongst gardens	0–7	0–13

Twenty live traps set per garden for five consecutive nights

Trapped animals were fur-clipped in order to avoid repeat counting

### Carabidae

Carabidae were trapped in 23 of the 28 gardens in the within-garden part of the study across the three sessions. One garden was excluded due to trap disturbance leaving 222 specimens, of which 220 could be identified to species (two were damaged). Eighteen species were found, with *P. madidus* dominating (*N* = 180). Nine species were represented by single individuals. See Table 3 for a complete species list.

**Table 3:** Total numbers of Carabidae caught in suburban domestic gardens in southern England (*N* = 22) provisioned with wild bird food in areas under a wild bird-feeding station (feeder pitfall traps) and a matched control further away

Species	Total in feeder pitfall traps	Total in control pitfall traps
*Agonum emarginatum*	1	0
*Abax parallelepipedus*	0	1
*Amara aenea*	2	1
*Amara convexior*	2	0
*Amara familiaris*	1	0
*Amara ovata*	1	0
*Amara similata*	1	0
*Bembidion properans*	1	0
*Calathus fuscipes*	1	4
*Calathus rotundicollis*	4	1
*Calathus melanocephalus*	0	2
*Harpalus latus*	6	1
*Harpalus affinis*	4	1
*Nebria brevicollis*	0	1
*Loricera pilicornis*	2	0
*Notiophilus biguttatus*	1	0
*Pterostichus madidus*	53	127
*Pterostichus strenuus*	1	0

Three pitfall traps were set in both the feeding and control areas in each garden.

**Fig. 1:**
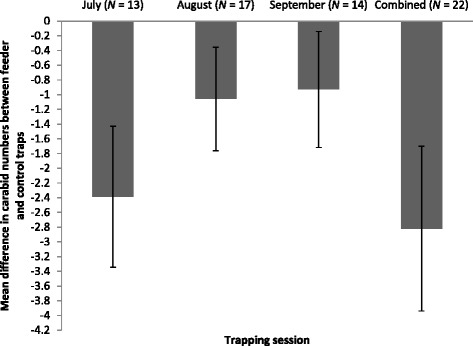
Mean differences ± SE in Carabidae numbers in pitfall traps in suburban domestic gardens in southern England in areas under wild bird-feeding stations and within-garden control areas of similar habitat away from feeders during monthly 1-week trapping sessions. *N* varies as carabids were not caught in all months in every garden. A paired *t-*test (combined data only; see text) revealed that the difference was significant (*t*_21_ = 2.52; *P* = 0.02)

Owing to the low power of the August and September data (0.29 and 0.19), all months were pooled (power = 0.67) prior to further analysis. However, Fig. 1 illustrates that the trend was for fewer carabids in the feeder traps compared with the controls in all months.

Significantly fewer carabids were caught in the feeder traps compared with the controls (means = 3.64 and 6.45, respectively; *t*_21_ = 2.52; *P* = 0.02; Fig. 1). The same pattern was found for *P. madidus* alone (means = 3.12 and 7.41; *t*_16_ = 3.55; *P* = 0.003) but not for all non-*P. madidus* combined (means = 2.06 and 1.75; *t*_15_ = 0.43; *P* = 0.672). The number of species was also not significantly different (*t*_21_ = −0.94; *P* = 0.359; means and ranges = 1.7, 0–6 and 1.2, 0–3, respectively). Owing to the low power of the latter two tests (0.07 and 0.15, respectively, vs. 0.64 for total numbers and 0.91 for *P. madidus*), Wilcoxon matched-pairs signed-ranks tests were also performed on these data and were also non-significant (non-*P. madidus* numbers: *P* = 0.776; Wilcoxon statistic = 74.0; median difference = 0.50; Q1–Q3 = −2.75–+2.00; species number: *P* = 0.438; Wilcoxon statistic = 52.5; median difference = 0.00; Q1–Q3 = −1.00–+1.00).

## Discussion

Our results broadly support two of our three hypotheses. Wild birds were significantly more likely to be recorded under bird-feeding stations than in the control non-feeding areas (with none in the control areas in all but one garden). Significantly fewer carabids were also trapped under feeding stations compared with to matched control areas although there was no significant differences in species number, perhaps owing to the relatively low numbers of species found per garden. In contrast to our predictions, we found no evidence of a difference in abundance of small mammals between gardens that provided wild bird food and those that did not. Overall therefore, our findings suggest a deleterious effect on local Carabidae numbers of the presence of bird feeders and of associated avian activity on the ground below.

Consistent with our initial hypothesis, our results show that ground-feeding birds are more likely to forage in areas under bird feeders than areas lacking feeders. We believe that the most likely explanation for the lower numbers of carabid beetles caught in these areas is predation by these birds. Carabidae form part of the diet of a large number of avian families, with birds even described as ‘among the most important species preying upon carabids’ (Thiele 1977: p. 93). Predation is consistent with the diets of many of the species recorded in the feeder areas. Magpie (*Pica pica*), blackbird, robin (*Erithacus rubecula*) and dunnock (*Prunella modularis*) diets in particular can contain considerable proportions of Coleoptera (dietary components for specific bird species are often only identified to order level). Values for magpies range from over 40 % in the UK to 65 % in Russia (Cramp et al. 1994). Specific percentages were unavailable for blackbirds (but see Cramp and Brooks 1988 for general dietary importance of Coleoptera for this species). The relatively small size and known diet of robins (Lack 1948) make it unlikely that a beetle as large as *P. madidus* (14–18 mm; Luff 2007) would be taken. However, faecal analysis of the slightly larger dunnock revealed 62 % Coleoptera by number, with 10 % of these being carabids [G. Bishton in Cramp and Brooks 1988 (data not published elsewhere)]. In work that classified Coleoptera to family level, the prevalence of Carabidae in the diet of these birds varied considerably (e.g., from 1 to 22 % of magpie diets; Cramp et al. 1994).

Even those birds recorded here that are not generally considered to be invertebrate feeders [collared dove (*Streptopelia decaocto*), chaffinch (*Fringilla coelebs*), woodpigeon, feral pigeon (*Columba livia*)] may occasionally consume Coleoptera (Cramp and Simmons 1980; Cramp and Brooks 1985, 1988; Cramp et al. 1994). For example, 3 % by number of chaffinch diet in Oxford during July to September (*c.* 30 km from Reading and matching our study season) was Coleoptera other than weevils (Newton 1967).

We also considered the alternative possibility that beetles avoided the areas under feeders due to disturbance from the significantly increased vertebrate activity relative to the control areas. As well as the birds described, grey squirrels (*Sciurus carolinensis*) were also frequently recorded but not analysed here as their diet is primarily plant based (weevils are the only known coleopteran component; Gurnell and Hare 2008). However, we consider this possibility unlikely because *P. madidus* (by far the most common carabid trapped: 180 specimens vs seven of the next most common, *Harpalus latus*) is predominantly active at night (Greenslade 1963; Thiele 1977). Therefore, its activity and/or movement patterns are unlikely to be greatly disturbed by vertebrate activity during daylight.

By contrast, we failed to find any evidence of a difference in either the abundance or species diversity of small mammals between bird-feeding and non-feeding gardens. Small mammal species diversity was not investigated statistically due to the very low numbers involved. We further speculate that the relatively low overall abundance of small mammals compared with the levels of bird activity in the within-garden work may mean that we lacked the statistical power to detect a difference. However, this also implies that any difference would necessarily be small. The low numbers in all categories also suggest that any contribution of mammalian predation would be slight compared with that of birds. It is relevant here that most traps remained unused on any given night in each garden, indicating that Longworth trap availability was not a limiting factor. We therefore suggest that birds are more likely than these mammals to be influencing carabid abundance in suburban gardens.

Earlier experimental studies by Martinson and Flaspohler (2003) and Orros and Fellowes (2012) provide further support for avian predation as an explanation. These authors attributed significant reductions in bark- and plant-dwelling arthropods, respectively, to predation by wild birds using feeders. The present study suggests a similar effect in a third habitat type. However, some difference in the spatial scale of the effect is evident. Martinson and Flaspohler (2003) found that depletion did not drop off over a 20-m radius from bird feeders. By contrast, the effect in the present study was highly localised; the within-garden feeder and control sites were just *c.* 4–15 m apart (depending upon garden size and location of matched habitats). In fact, the garden with the shortest distance had the second greatest difference in carabid numbers (data not shown). Although the range of depletion remains unknown for aphids [Orros and Fellowes (2012) used 1 m distances only], the range of visible influence clearly varies amongst some prey taxa.

The spatial scale at which any ecological effect acts is key to understanding its biological significance. We speculate that the very local effect in the present study indicates that areas of gardens may act as refugia from avian predation even if wild birds are regularly fed. Furthermore, it raises the intriguing possibility that gardens in which birds are not fed (1/3 of domestic gardens in England; ODPM 2003) may also act as carabid refugia. Such gardens could easily be assumed not to have high wildlife value given that wild bird feeding is the most popular form of ‘wildlife gardening’ in the UK (Gaston et al. 2007). However, the effectiveness of some popular wildlife gardening methods is equivocal (Gaston et al. 2005) and further, some otherwise keen wildlife gardeners may not feed birds for reasons such as cat presence (R. L. Thomas, pers. observ.).

Although the predominant carabid species found here, *P. madidus*, is extremely common in Britain and elsewhere (Luff 2007) and of little conservation significance, many rare arthropods have been recorded in domestic gardens in urbanized areas (Owen and Owen 1975; Owen 1991). Bird-feeding stations or other point attractants, such as nesting sites or fruit/berry-bearing plants, could therefore have the potential to adversely affect small or local populations of palatable prey species, especially if several are present. It is interesting in this context that we found no carabids in five gardens, and that half of the species were represented by single individuals. Although these findings may be at least partly due to survey effort and sample size, pitfall trapping is generally regarded as an effective (and widely tested) survey method for many Carabidae including those found here (e.g., Greenslade 1964). Therefore, this result suggests that some species may exist in very low numbers in domestic gardens and therefore could be adversely affected by even very localised depletion.

Further research worldwide is required to investigate whether the effects observed here on carabids and previously on aphids (Orros and Fellowes 2012) in English suburban gardens and those seen in North American woodland (Martinson and Flaspohler 2003) extend across other similar and different habitats, and to other species and seasons. We speculate that research to date has hardly scratched the surface of the possible indirect influences of wild bird feeding, particularly given the vast scale of the activity in many countries.

## Conclusion

This study provides the first evidence that supplementary feeding of wild birds in private domestic gardens can deleteriously affect numbers of a common ground-living arthropod taxon. We have also shown that such indirect effects of garden bird feeding can occur under householders’ usual bird-feeding habits. Three arthropod taxa with differing habitat requirements are now known to be negatively influenced by anthropogenic feeding of wild birds.
